# Editorial for the Special Issue “Epidemiology of Enterovirus Disease”

**DOI:** 10.3390/microorganisms10061221

**Published:** 2022-06-15

**Authors:** Antonio Piralla, Elena Pariani

**Affiliations:** 1 Microbiology and Virology Department, Fondazione IRCCS Policlinico San Matteo, 27100 Pavia, Italy; 2 Department of Biomedical Sciences for Health, University of Milan, 20133 Milano, Italy; elena.pariani@unimi.it

Enteroviruses (Enterovirus genus, *Picornaviridae* family) are distributed worldwide and are among the most common causes of human disease globally. This viral genus includes both well-known human pathogens—such as poliovirus, coxsackievirus and rhinoviruses—and emerging viruses—such as enteroviruses 71 and D68. Enteroviruses (EVs) are associated with a wide variety of clinical manifestations, ranging from respiratory, gastrointestinal or skin symptoms to severe infections in the myocardium or central nervous system. The number of new EVs has been increasing in recent years, and more than 250 genotypes, including 160 rhinoviruses (RVs) genotypes, have been recognized so far. Emerging EVs, such as D68 and EVs belonging to species C, have taught us that even in a polio-free world, these viruses are still able to cause devastating diseases, such as severe neurological infections, polio-like acute flaccid paralysis/myelitis and life-threatening respiratory diseases. In this perspective, EV surveillance takes on a key role in the post-polio elimination era, acting as an early warning system, not only of any potential re-introduction of poliovirus but also of outbreaks of other potentially threatening enteroviruses, as demonstrated by the re-emergence of EV-D68 in North America and some European countries over the last few years. The overall aim of this Special Issue is to provide a global and comprehensive picture of EVs’ circulation, epidemiological characteristics and changes in clinical presentations associated with either unexpected or well-known EV genotypes that may have an impact on public health. This Special Issue includes a review [1] and a research article [2] describing the impact of rhinoviruses as pathogens involved in bronchiolitis or as a cause of asthma exacerbations in both children and adults [1], as well as a cause of prolonged infections in immunocompromised patients [2]. Other articles focus on the circulation of EVs among human and great apes [3] and on Echovirus 30 (E30) as a cause of neurological disorders [4]. Finally, an article authored by the European Non-Polio Enterovirus Network (ENPEN) presents a complete overview of EVs’ circulation and the estimation of the disease burden of EV and human parechovirus (PeV) infections in Europe through the establishment of standardized syndromic surveillance for hand, foot and mouth disease (HFMD) and respiratory and neurological infections.

A summary of the most recent findings on the role of RV infection in children with acute bronchiolitis, its impact on subsequent asthma development and the implications in clinical practice are discussed in the article by Biagi et al. [1]. Acute bronchiolitis represents the leading cause of hospitalization in infants, and RVs, together with respiratory syncytial virus, are the most common pathogens associated with bronchiolitis. RVs’ genetic diversity (>150 types) makes the recurrence of RV infections quite typical. The frequency of RV infections and co-infections with other viruses and their impact on the clinical course of bronchiolitis have been studied by several researchers with controversial results. Some studies have demonstrated that multiple virus infections result in more severe clinical presentation and a higher risk of complications, whereas other studies have suggested no influence on clinical course. Moreover, RV bronchiolitis has been reported to potentially contribute to the development of long-term sequelae, such as recurrent wheezing and asthma, in the pediatric population.

The impact of RVs in the hospital setting is presented in the article by Giardina et al. [2], describing the results of a 15-month surveillance of RVs’ circulation in Italy. In this study, the median age of RV/EV-positive patients is 9 years; RV species A and C were detected in the majority of cases, while RV species B accounted for less than 10% of cases. A total of 7% of the patients included in this study had a prolonged infection with a median duration of 62 days: all these patients were immunocompromised, and most of them were children with an RV-A infection. Two outbreaks were identified, one in the neonatal intensive care unit and one in the oncohematology department, and were caused by RV-A89 and RV-C43, respectively. Nearly 5% of patients—all of whom had preexisting comorbidities—were admitted to the intensive care unit and required mechanical ventilation [2].

EVs may cause mild to severe infections in humans and in several animal species, including non-human primates. The study by Amona et al. [3] describes the results of the characterization of EVs circulating between humans and great apes in the Congo. Fecal samples (N = 24) of gorillas and chimpanzees living close to or distant from humans in three Congolese parks were collected, along with fecal samples from healthy humans (N = 38) living around and within these parks. EVs were detected in 29.4% of gorilla feces and in 13% of human feces. Two identical strains were isolated from two humans coming from two remote regions. Their genomes were similar, and all genes showed their close similarity to coxsackieviruses, except for the 3C, 3D and 50-UTR regions, where they were most similar to poliovirus 1 and 2, suggesting a possible event of recombination. Recombination events were found between these strains, poliovirus 1 and 2 and EV-C99. It is possible that the same EV species C circulated in both humans and apes in different regions of Congo. Of course, further investigations on the circulation and genetic diversity of EVs in the population of great apes are needed to draw a clearer picture on the different species and types of EVs circulating in the Republic of Congo [3].

Several EVs were described as causative agents of neurological disorders. Echovirus 30 (E30) is one of them, and multiple studies of outbreaks have been published, but only a few assessed E30 epidemiology over a long period of time. The article by Del Cuerpo et al. [4] presents the results of the analysis of the clinical, epidemiological and microbiological characteristics of a series of E30 infections detected over the last 26 years. E30 was detected by viral isolation or nucleic acid detection in patients presenting with respiratory or neurological infections, rash, sepsis-like syndrome or gastroenteritis. Of the 2402 EV infections detected, 1619 were linked to at least one genotype, and 173 were caused by E30. Clinical information was available for 158 (91.3%) patients. E30 was associated with neurological infection in 107 (67.8%) cases, and it was detected almost every year. Phylogenetic analysis performed with 67 sequences showed that E30 strains belonging to two lineages (E and F) circulated in Catalonia from 1996 to 2016. In 2018, lineage I emerged as the dominant lineage [4].

Finally, the European Non-Polio Enterovirus Network (ENPEN) presents the results of a prospective, multi-center and cross-sectional hospital-based pilot study on EVs developed through standardized protocols [5]. Limited data on their true disease burden of EVs exist, as standardized European-wide surveillance is lacking. The aim of this network is to estimate the disease burden of EV and PeV infections in Europe via the establishment of standardized surveillance for hand, foot and mouth disease (HFMD) and respiratory and neurological infections caused by these viruses. A comparison of assays used in different laboratories in terms of sensitivity was implemented in the network of participating laboratories so that all EV and PeV types are adequately detected. The plan of sharing protocols include guidance for diagnosis, case definition, detection, characterization and reporting of EV and PeV infections associated with HFMD and respiratory and neurological diseases has been presented. Over 30 sites from 17 European countries have already registered to this one pilot study, likely to be commenced in 2022. This surveillance will allow for a European-wide comparison of data on EV and PeV infections. These data will also be used to determine the burden of EV and PeV infections, which is needed to guide further prevention measures and policies [5].

In conclusion, the original articles and review published in this Special Issue give a comprehensive overview on *Picornaviridae*, with interesting illustrations of the importance of improving and increasing our knowledge on this field.
